# Caregiver Social Status and Health-Related Quality of Life in Neurologically Impaired Children on Home Enteral Nutrition

**DOI:** 10.3390/nu13061928

**Published:** 2021-06-04

**Authors:** Valeria Dipasquale, Marco Ventimiglia, Simone Maria Calogero Gramaglia, Barbara Parma, Caterina Funari, Angelo Selicorni, Chiara Armano, Silvia Salvatore, Claudio Romano

**Affiliations:** 1 Pediatric Gastroenterology and Cystic Fibrosis Unit, Department of Human Pathology in Adulthood and Childhood “G. Barresi”, University of Messina, 98124 Messina, Italy; dipasquale.valeria@libero.it (V.D.); simone.gramaglia10@gmail.com (S.M.C.G.); 2 Inflammatory Bowel Disease Unit, “Villa Sofia-Cervello” Hospital, 90146 Palermo, Italy; marco20miglia@gmail.com; 3 Department of Pediatrics, Sant’Anna Hospital, 22042 Como, Italy; barbaraparma79@hotmail.com (B.P.); caterina.funari@virgilio.it (C.F.); angelo.selicorni61@gmail.com (A.S.); 4 Pediatric Department, “F. Del Ponte” Hospital, Insubria University, 21100 Varese, Italy; chiara.armano86@gmail.com (C.A.); silvias.varese@gmail.com (S.S.)

**Keywords:** children, enteral nutrition, health-related quality of life, home enteral nutrition, neurological impairment, social status

## Abstract

We aimed to investigate the association between caregiver social status and health-related quality of life (HRQoL) in children with neurological impairment (NI) on home enteral nutrition (HEN). This was an ancillary study of a multicenter, cross-sectional study which explored HRQoL in 75 NI children on HEN. All the caregivers from the original cohort were contacted, and data on education level, occupation and marital status were collected. The association between social status and HRQoL was investigated using a multiple Poisson Generalized Linear Model. In total, 93 caregivers were included, responsible for the care of 71 children. The caregivers of four children of the original cohort did not answer the questionnaire. Mothers with high-level education presented lower HRQoL in comparison to mothers with low-level (β: −5.97; 95% CI −11.51, −0.10; *p* = 0.027) or medium-level education (β: 4.85; 95% CI −9.87, 0.53; *p* = 0.044). The analysis of the subgroup of cases in which the main caregiver was represented by both parents gave similar findings, with education level of the father being negatively correlated with HRQoL. Our data showed that higher education level may negatively affect quality of life of caregivers of NI children. This could be helpful in identifying at-risk families and addressing supportive efforts.

## 1. Introduction

Children with neurological impairment (NI) often experience reduced health-related quality of life (HRQoL), mainly due to feeding and gastrointestinal problems, which result in faltering growth and malnutrition. In general, quality of life is defined as the perceived quality of an individual’s daily life, including all emotional, social and physical components. In health care, HRQoL is the assessment of how a disease, disability or disorder may affect the individual’s well-being [1,2], and it is recognized as the most relevant outcome for chronic conditions such as NI [3]. Children with severe NI are not able to self-report their sensations of their quality of life; therefore, parent-proxy reports are the only available measures. It has been largely shown that caregivers of children with NI have worse mental health, higher burnout levels and very low quality of life [4]. Over the last few years, it has been shown that enteral nutrition (EN) is effective in reversing malnutrition and positively affecting HRQoL in children with NI, even when administered at home [5,6,7]. Indeed, EN is usually started in the hospital, but the lifelong duration of many programs requires home enteral nutrition (HEN), thus preventing complications and high costs due to prolonged hospitalization [8]. Neurological disabilities are the first indications for enteral tube feeding and HEN in childhood [9,10]. A significant improvement in social functioning, mental health, vitality, and general health perception after starting EN has been reported [11,12]. Higher HRQoL levels in children with NI after tube feeding were verified in lots of studies in the literature, and the factors influencing HRQoL have also started to be investigated. Social status is a composite measure including educational attainment, employment and marital status, and it is indicative of an individual’s social position [13,14]. Family social status is considered an important factor influencing an individual’s neural and cognitive development [15,16,17]. For example, in young individuals, higher family social status seems to be associated with better cognitive and memory functions, including better working memory, executive function and language skills [18,19]. Higher family social status is also associated with increased self-regulatory behaviors, a greater sense of well-being and less psychological distress in young individuals [20,21,22,23]. Currently available literature data suggest that social status may significantly affect the quality of life in children with chronic conditions [24,25,26], due to many factors, such as the impact of financial resources and/or education level on health services utilization, unplanned readmission, difficulty with medication instructions, and post-discharge survival. A recent systematic review assessed the association between socio-economic disadvantage and quality of life among children with common chronic conditions (asthma, epilepsy, type 1 diabetes mellitus and chronic kidney disease) [24]. A total of 22 studies found a statistically significant association between at least one socioeconomic determinant (education, occupation, marital status, income and health insurance coverage) and lower quality of life in paediatric patients with one of the above-mentioned chronic diseases [24]. In our recent cross-sectional study of HEN-related quality of life in NI children [27], the total scores of quality of life were found to be significantly associated with the underlying disease (children with neurological disorders had higher HRQoL scores than those with genetic or metabolic diseases, *p* = 0.014) and marginally associated with HEN duration (scores decreasing by 0.19 points for each year spent on HEN, *p* = 0.098). In our previous study, it was not explored caregiver social status and its potential influence on HRQoL. The aim of the present study was to investigate the association between caregiver social status and HEN-related quality of life in children with NI.

## 2. Materials and Methods

### 2.1. Participants

The present study was an ancillary study of a multicenter, cross-sectional study based in Italy. Briefly, the multicenter, cross-sectional study included 80 children with NI on HEN who were followed in 3 tertiary hospitals between January 2013 and September 2018 [18]. The inclusion criteria comprised: (a) age ≤ 18 years; (b) progressive or nonprogressive NI; (c) exclusive enteral feeding; (d) ≥12-month duration on HEN. Neurological impairment referred to a heterogeneous group of disorders that primitively relate to the central nervous system, made up of the brain and spinal cord, affecting an individual’s speech, motor abilities, vision, memory, muscle actions, and learning skills. Primary caregivers were invited to complete, via telephone, an original survey made up of 12 questions on HRQoL that were strictly related to HEN, designed by a multidisciplinary team made up of paediatricians, paediatric gastroenterologists and dietitians. It encompassed items of physical functioning (i.e., the easiness of feeding the child, weight, physical discomfort, respiratory symptoms and HEN management) and psychological and social dimensions (i.e., daily activities, mealtimes, sleep patterns and the ability to go out with friends).

From October 2019 to December 2019, all the caregivers of the 75 NI children from the original cohort were contacted to be enrolled in this ancillary study. The present study was carried out in conformity with the principles and regulations of the Helsinki Declaration. Informed consent was obtained from the patient’s parents or legal guardians, who were fully informed of the nature and purpose of the study. The protocol of the study was approved by the Institutional Ethics Committee of Messina University Hospital.

### 2.2. Data Collection

For the purpose of the present study, we updated information on education level, occupation and marital status of each caregiver at the time of initial study entry during telephone interview. For each caregiver, education was categorized as low (less than high school), medium (high school or equivalent) and high (college graduate or above). Occupations included unemployed/housewife, manual worker or farmer (level 1), trader or clerk (level 2), businessman, teacher, physician, lawyer or freelancer (level 3). Marital status encompassed married, divorced and widow(er). All the updated information were reported by caregivers and collected through a researcher-designed data collection form. The results were analyzed statistically according to HRQoL scores.

### 2.3. Statistical Analysis

Continuous variables were reported as medians with ranges, and categorical variables were reported as frequencies and percentages. A multiple Poisson Generalized Linear Model (GLM) was used to determine the association between the HRQoL score and possible predictors. In the univariate analysis, caregivers were divided into three categories (mothers, fathers and grandmothers). In the multivariate analysis, with the mother being the caregiver (alone or with the father) in most cases, the main analysis focused on the evaluation of the impact of maternal social status on HRQOL score. A subgroup analysis including the cases in which both parents (mother + father) were caregivers was performed in order to evaluate the association between the social status and total HRQOL scores. Variable selection was performed using a stepwise backward elimination approach, based on the Akaike information criterion. GLM diagnostics were performed by residual analysis and the Generalized Variance Inflation Factor (GVIF) was used for the assessment of multicollinearity: no violation of model assumptions was found.

## 3. Results

Seventy-five (93.75%) surveys were completed in the original cohort study, and the total scores are reported in Table 1.

A total of 93 caregivers from 71 unrelated families were included in the present study. These 93 caregivers were responsible for the care of 71 paediatric patients on HEN. The caregivers of four children of the original cohort did not answer the questionnaire. In 44 cases (62.0%), the main caregiver was the child’s mother; in 22 cases (31.0%), the caregivers were the child’s mother and father; in four cases (5.6%), the caregiver was the child’s grandmother; and in 1 case only (1.4%), the caregiver was the child’s father. The median age of the mothers was 29 years (range: 20–55 years); the median age of the fathers was 33 years (range: 22–50 years). The mean Short Portable Mental Status Questionnaire score was 0.5. The social status of all participants is illustrated in Table 2.

Regarding the education level, the majority of mothers (48.5%) reported medium-level education, while most fathers and grandmothers had low-level education. The majority of caregivers in each category had an occupation included in level 1 (83.3% of mothers, 69.6% of fathers and half of the grandmothers). Nearly all (*n* = 80) the caregivers were married.

**Figure 1:**
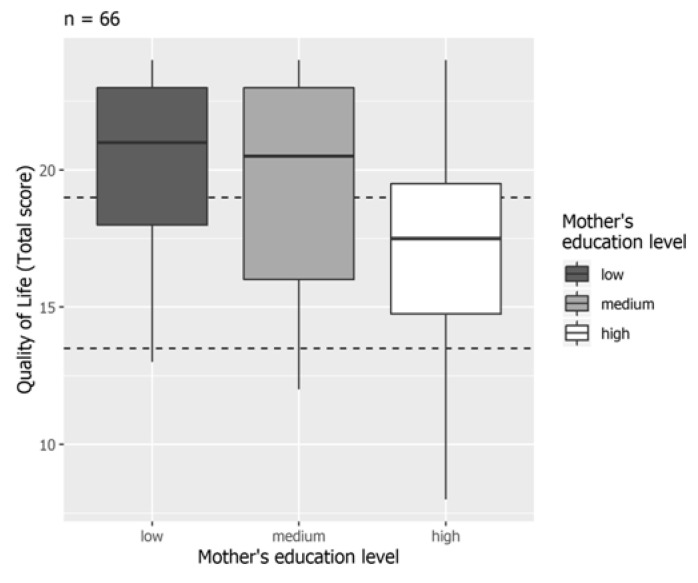
Health-related quality of life and education level of mothers.

Due to the small number of grandmothers, univariate and multivariate analyses included mothers and fathers only. Univariate analysis did not find any association between HRQoL and either education level (*p* = 0.294 for mothers, *p* = 0.217 for fathers) or occupational status (*p* = 0.188 for mothers, *p* = 0.618 for fathers). Similarly, marital status did not influence the HRQoL (*p* = 0.605 for mothers, *p* = 1 for fathers). Despite no significant association in the univariate analysis, in the multiple Poisson regression model analysis, a significant negative correlation between HRQoL and the education level of all mothers was found: mothers with high-level education were associated with lower HRQoL scores in comparison to mothers with low-level (β: −5.97; 95% CI −11.51, −0.10; *p* = 0.027) or medium-level education (β: −4.85; 95% CI −9.87, 0.53; *p* = 0.044) (Table 3; Figure 1).

**Figure 2:**
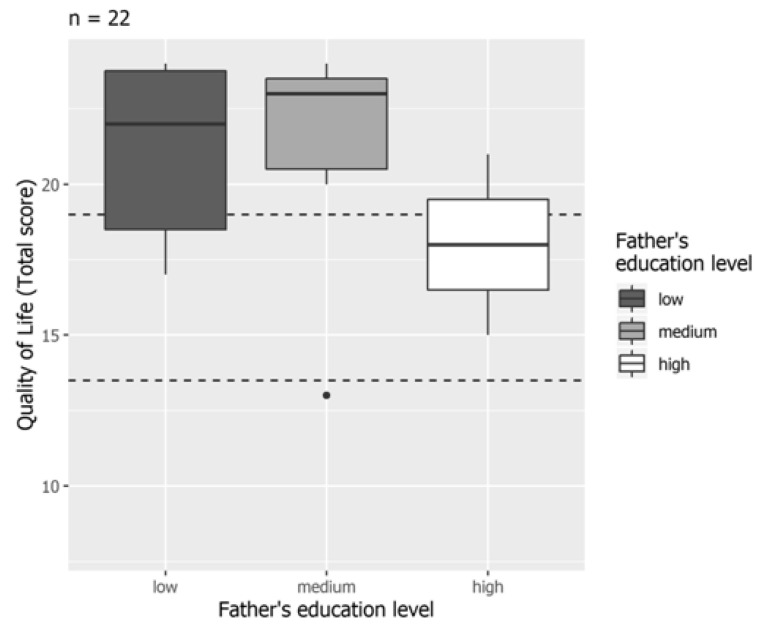
Health-related quality of life and education level of fathers in the subgroup of children with both parents as caregivers.

The multivariate analysis of the subgroup of cases in which the main caregiver was represented by both parents (*n* = 23) gave similar findings. There was a significant negative correlation between HRQoL and the education level of one of the two parents (the father): fathers with high-level education were associated with lower HRQoL scores in comparison to fathers with low-level (β: −6.28; 95% CI −10.69, −1.58; *p* = 0.017) or medium-level education (β: −5.50; 95% CI −9.67, −1.00; *p* = 0.025) (Table 4; Figure 2).

In this subgroup, no mother had a high level of education and, for this reason, no significant association between mothers’ education level and HRQoL was found.

## 4. Discussion

Our findings suggest that social status may be a determinant of HRQoL in children with NI on HEN. To the best of our knowledge, this is the first study exploring the association of social status and HEN-related quality of life in this group of children. Different studies showed that social and economic status may affect the quality of life in children with chronic disease [24]. The association between social status and HRQoL in children with neurodisability has been scarcely investigated and available data are conflicting. An Iranian study aimed to compare the quality of life of mothers of paediatric patients with cerebral palsy (*n* = 120) with that of mothers of typically developing children (control group; *n* = 100) showed that quality of life was lower in the first group, and significantly deteriorated by lower levels of socioeconomic status (including education level, occupational status, income and housing situation), higher amounts of marital dissatisfaction and higher levels of perceived fatigue [28]. It is important to highlight that the accessibility to free transportation services in western families may further decrease the impact of socioeconomic status on HRQoL. Most parents also receive a monthly financial grant to compensate for expenses, which may decrease extreme stress and financial burden in families from the lowest socioeconomic groups [28]. No association was found between marital status and HRQoL [28]. Accordingly, other studies reported a socioeconomic gradient for cerebral palsy [29,30,31]. In a study by Raina et al. [32], higher socioeconomic status was associated with fewer psychological life stressors, better emotional functioning, fewer child behavior disorders and better physical health. However, Blacher et al. [33] reported that social status cannot be considered as a buffer against depression in the presence of specific stressors such as neurological disorders. Conversely, no association between HRQoL of mothers of children with cerebral palsy and their social status was found [34,35]. A cross-sectional survey conducted in Nigeria, West Africa, enrolled 80 mothers—40 of children with cerebral palsy and 40 of age-matched typically developing children [36]. No significant influence of educational and occupational status on the quality of life scores of mothers of children with cerebral palsy (which was significantly lower than quality of life scores of mothers of typically developing children) was showed. Variability among studies could be influenced by the geographical and cultural differences among populations, directly affecting the social status of caregivers. Moreover, studies that report socioeconomic status often use various methods of description [37]. However, none of these studies provide information on whether NI children were on EN or not. In the present study, the social status indicator that was showed to influence the HRQoL was education level, with higher-level education being associated with lower total quality of life scores. Living with a chronic disease not only affects the child but also the other members of the child’s family, especially the primary caregiver, who often is the child’s mother and/or father. The commitment to long-term care for these children often requires them to sacrifice some of their own life projects and expectations in order to devote large amounts of time to care for their children. This could be true especially for those caregivers who spent a lot of time in education, who have to rearrange their priorities and redirect their energies, likely worsening their burden and stress. Moreover, a higher educational level, while helping in developing compensation strategies to overcome the child’s disability, may also increase the awareness of the child’s chronic and often progressive condition. Identifying the factors affecting quality of life can help in identifying patients and families to be addressed with closer, more intensive psychological support, and enhance the overall care and rehabilitation programs. Further studies are warranted, also to consider the presence of additional older children as well as other adults in the home who could help to share some of these responsibilities. Conversely, additional younger children could add to caregiver burden and stress, thus making measures of HRQoL appear worse.

Notably, in the present study, a quantitative prevalence of mothers as primary caregivers was observed. However, a good portion of caregivers was represented by both the mother and father, differently to previous cohorts. In most parts of the world, women have more responsibility in raising children, and the primary caregivers of children with chronic diseases and disabilities are usually mothers [35]. In facing the problems of children, mothers have been shown to be more affected than fathers [37,38].

The present study has some limitations. First, it is a retrospective study; therefore, recruited cases and clinical management represent the confounding variables. Moreover, the survey was administered by phone, and this could have introduced a bias (i.e., time-constrained interviews, hard-to-reach respondents or lack of representativeness). Second, the study did not explore the influence of variables such as the age of caregivers, their income or social support, which had been reported to affect quality of life. Third, we did not differentiate among HRQoL scores of each underlying disease. The main strength of the present study is that it is one of the few studies to explore the influence of social status on HRQoL in NI children living in Europe, and the first study to explore this association in children with NI on HEN.

In conclusion, the present survey investigated the effect of caregiver social status on HEN-related quality of life of NI paediatric patients. The main finding was that higher education levels may negatively affect the quality of life of caregivers. This could help healthcare providers to identify at-risk families and to better address supportive efforts and resources.

## Figures and Tables

**Table 1:** Total scores of health-related quality of life from the original cohort study.

Total Score	HRQoL	Total, *n* = 75
20–24	Excellent	47 (62.7%)
14–19	Good	22 (29.3%)
7–13	Acceptable	6 (8.0%)
0–6	Poor	0

HRQoL, Health-related quality of life.

**Table 2:** Social status of caregivers.

Variable	Mothers, *n* = 66	Fathers, *n* = 23	Grandmothers, *n* = 4
Education level			
Low	30 (45.4%)	14 (60.9%)	2 (50.0%)
Medium	33 (48.5%)	7 (30.4%)	2 (50.0%)
High	4 (6.1%)	2 (8.7%)	0
Occupation level			
Level 1	55 (83.3%)	16 (69.6%)	2 (50.0%)
Level 2	8 (12.1%)	6 (26.1%)	1 (25.0%)
Level 3	3 (4.6%)	1 (4.3%)	1 (25.0%)
Marital status			
Married	57 (86.4%)	21 (91.3%)	2 (50.0%)
Divorced/separated	8 (12.1%)	2 (8.7%)	2 (50.0%)
Widow(er)	1 (1.5%)	0	0

**Table 3:** Multiple Poisson regression model estimates for health-related quality of life score. Results for main analysis (mother as caregiver, alone or with father).

Variable	β	(95% CI)	*p*
(intercept)	13.02	(6.72, 19.75)	<0.001
Neurological disease (ref = genetic)	3.09	(1.04, 5.11)	0.004
Metabolic disease (ref = genetic)	1.19	(−2.38, 4.97)	0.528
Caregiver both parents (ref = only mother)	1.55	(−0.48, 3.60)	0.141
Age at hen beginning (years)	0.05	(−0.21, 0.32)	0.708
HEN duration (years)	−0.24	(−0.50, 0.01)	0.065
Mother’s occupation level 2 (ref = level 1)	0.70	(−2.66, 4.16)	0.690
Mother’s occupation level 3 (ref = level 1)	4.50	(−1.42, 10.65)	0.113
Mother’s education level low (ref = high)	5.97	(0.10, 11.51)	0.027
Mother’s education level medium (ref = high)	4.85	(−0.53, 9.87)	0.044

HEN, home enteral nutrition.

**Table 4:** Multiple Poisson regression model estimates for health-related quality of life score. Results for subgroup analysis (both parents as caregiver).

Variable	β	(95% CI)	*p*
(intercept)	19.52	(16.80, 22.34)	<0.001
Neurological disease (ref = genetic)	5.07	(2.38, 7.70)	0.002
Metabolic disease (ref = genetic)	2.21	(−0.78, 5.21)	0.173
HEN duration (years)	−0.27	(−0.52, −0.01)	0.058
Father occupation level 2 (ref = level 1)	−0.63	(−3.50, 2.28)	0.682
Father occupation level 3 (ref = level 1)	6.17	(−0.19, 12.61)	0.081
Father’s education level low (ref = high)	6.28	(1.58, 10.69)	0.017
Father’s education level medium (ref = high)	5.50	(1.00, 9.67)	0.025

HEN, home enteral nutrition.

## Data Availability

The data presented in this study are available on request from the corresponding author.
